# Supplementary material for: Relative Biological Effectiveness of Carbon Ions for Head-and-Neck Squamous Cell Carcinomas According to Human Papillomavirus Status
Source: J Pers Med. 2020 Jul 25;10(3):71. doi: 10.3390/jpm10030071 (PMC7565683; doi:10.3390/jpm10030071)
Supplement: Supplementary file 1 [file jpm-10-00071-s001.pdf]

Table S1. α and β parameters for X-rays or carbon ions for HPV-possitive and -negative lines.

Cell line	HPV status	X-rays		Carbon ions	
		α	β	α	β
UD-SCC-2	Positive	0.10	0.080	0.82	0.043
UM-SCC-47	Positive	0.42	0.019	0.046	0.49
UM-SCC-104	Positive	0.19	0.027	1.21	-0.060
UPCI:SCC154	Positive	0.72	-0.010	0.67	0.11
A-253	Negative	0.43	-0.0090	0.73	0.0090
Detroit 562	Negative	0.17	0.0050	0.32	0.073
FaDu	Negative	0.13	0.012	0.66	0.016
SCC-9	Negative	0.44	0.018	0.55	0.010
SCC-25	Negative	0.27	0.036	0.75	0.098
UM-SCC-1	Negative	0.12	0.013	0.57	0.062

HPV, human papillomavirus; HNSCC, head and neck squamous cell carcinoma.

Figure S1

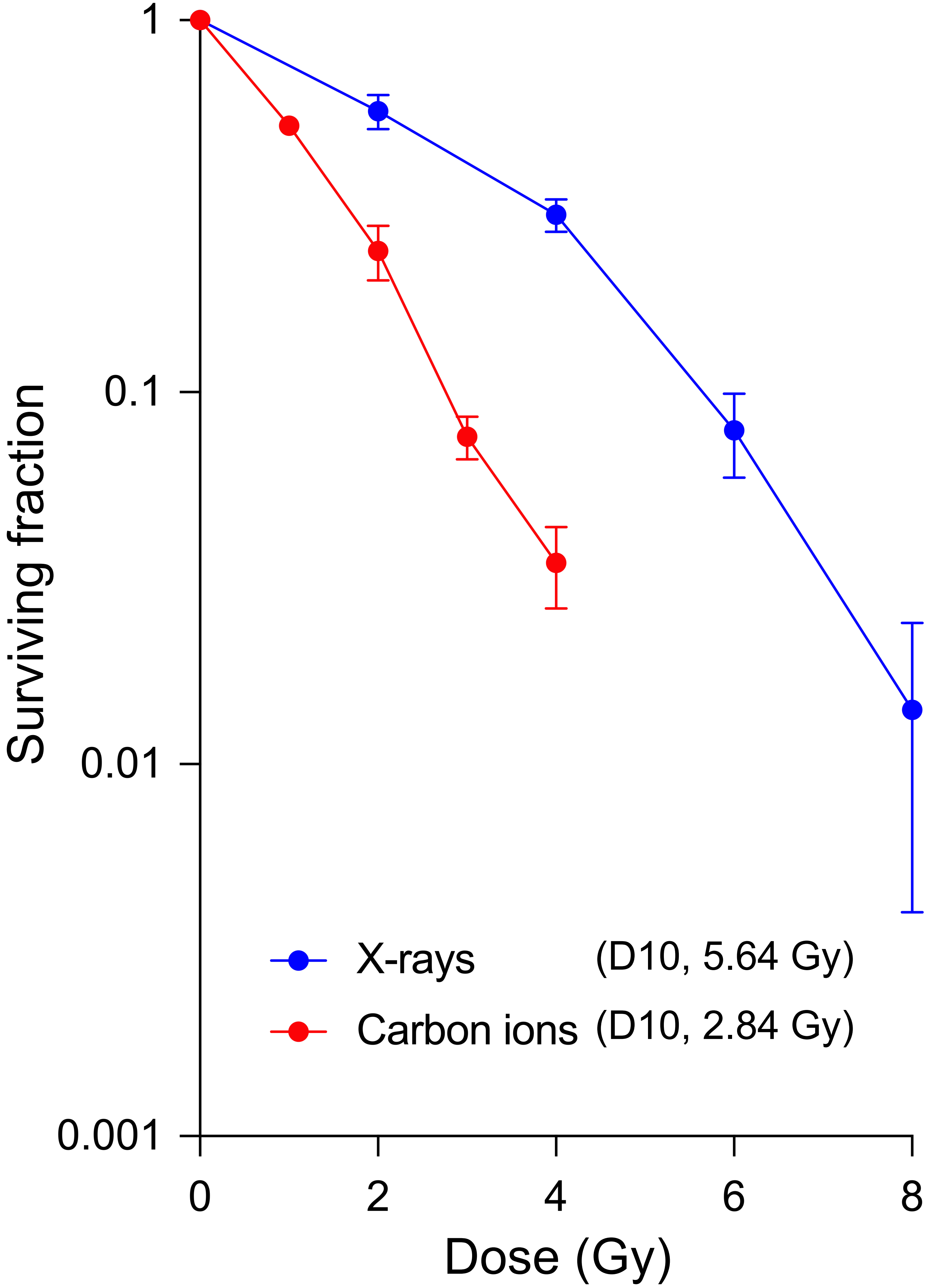
1
0.1
0.01
0.001
Surviving fraction
0
2
4
6
8
Dose (Gy)
X-rays (D10, 5.64 Gy)
Carbon ions (D10, 2.84 Gy)
